# Enhancing Clinical Competencies Through Peer Role-Play in Oncology Graduate Students: Mixed Methods Study

**DOI:** 10.2196/79771

**Published:** 2025-11-28

**Authors:** Yao Wang, Feixiang Wang, Gaojie Liu, Yuqing luo, Hongjun Ba, Jie Long

**Affiliations:** 1 Guangzhou Medical University Cancer Hospital Guangzhou, Guangdong Province China; 2 Department of Pediatrics The First Affiliated Hospital, Sun Yat-sen University Guangzhou, Guangdong Province China

**Keywords:** peer role-play, Mini-Clinical Evaluation Exercise, clinical competencies, oncology, physician-patient communication

## Abstract

**Background:**

Clinical competency is essential for oncology students to deliver high-quality patient care. However, traditional teaching methods may not fully support the development of critical skills such as communication, empathy, and clinical judgment. Peer role-play has emerged as a promising approach to bridge these gaps by enhancing interpersonal and diagnostic competencies within clinical settings.

**Objective:**

This study aims to evaluate the effectiveness of peer role-play in developing clinical competencies among oncology graduate students during their clinical rotation.

**Methods:**

This study involves 70 first-year oncology graduate students from Affiliated Cancer Guangzhou Medical University Cancer Hospital in a 3-month clinical rotation within the department of oncology from January 2022 to December 2023. Participants were randomly assigned to either a peer role-play group (n=35) or a traditional teaching group (n=35), ensuring balanced gender and baseline competencies. The role-play group engaged in a structured curriculum that included case presentation, classroom instruction, and weekly role-play sessions, with debriefing and feedback sessions following each role-play. The traditional teaching group adhered to a standard curriculum without role-play exercises. Assessments included a baseline oncology theory exam, Mini-Clinical Evaluation Exercise for clinical competency evaluation, and a satisfaction survey for the role-play group.

**Results:**

Baseline theory exam scores were comparable between the 2 groups (*P=*.08). However, the peer role-play group demonstrated significant improvements in doctor-patient communication, medical history taking, clinical judgment, and overall clinical competence compared to the traditional teaching group (*P*<.05). Furthermore, students in the role-play group reported high levels of satisfaction, citing scenario realism, communication practice opportunities, and feedback quality as key benefits.

**Conclusions:**

This study indicates that peer role-play is an effective educational approach for developing clinical competencies in oncology graduate students, particularly in communication, empathy, and clinical reasoning. Role-play provides an engaging and practical learning experience, making it a valuable addition to clinical training programs aimed at enhancing patient-centered care skills in students.

## Introduction

### The Need for Effective Communication in Oncology Education

The rapid advancements in medical knowledge and technology have reshaped oncology practice, increasing the demand for oncology graduate students to develop clinical competencies and patient-centered communication skills [1,2]. In oncology, where patients face emotionally and physically challenging conditions, the ability to communicate with empathy, clarity, and sensitivity is essential. Discussions involving complex diagnoses, treatment options, and prognoses have a profound impact on patients and their families, requiring clinicians to balance clinical accuracy with emotional awareness. However, mastering these communication skills can be challenging for students newly entering clinical settings. Many lack the experience to navigate oncology’s intense emotional landscapes, making it difficult to build rapport, demonstrate compassion, and manage challenging conversations [3].

Traditional medical education, largely based on lectures and observational learning, often falls short in fostering the interpersonal skills necessary for these interactions. There is a critical need for training approaches that provide early, practical opportunities for realistic patient engagement. Role-play, an active learning methodology, offers a promising solution by enabling students to assume both practitioner and patient roles within controlled simulations [4]. This technique allows students to practice essential clinical skills in a low-risk setting and helps them develop effective, empathetic communication with patients. By simulating real-life interactions, role-play encourages students to develop the confidence and sensitivity required to manage emotionally complex oncology conversations before they enter clinical practice [5].

Despite the recognized importance of communication in oncology, few studies have examined the specific impact of role-play on preparing students for these complex interactions. Most research on role-play in medical education focuses on general skill development or communication in less specialized fields [6], leaving a gap in understanding its potential in comprehensive oncology training. This study sought to bridge this gap by investigating the effectiveness of role-play in enhancing oncology graduate students’ clinical competencies, especially in patient communication, diagnostic reasoning, and emotional intelligence.

### Role-Play as a Tool for Enhancing Patient-Centered Communication

Role‑play can be situated within an experiential learning and deliberate practice framework, allowing learners to iteratively rehearse complex communicative and diagnostic tasks with targeted feedback in a psychologically safe environment. In medical education, role‑play has been used both informally and within structured curricula to improve communication, empathy, and professionalism, with guidance on task design, briefing, and debriefing to maximize learning [7]. Peer role‑play specifically offers a low‑cost, scalable alternative to standardized patients (SPs) and high‑fidelity simulation while retaining core pedagogical features such as scenario authenticity, role clarity, and structured feedback [8]. Our study extends this literature by testing a systematic, oncology‑specific peer role‑play curriculum during a real clinical rotation.

In oncology, communication frequently involves serious illness conversations, including breaking bad news and discussing goals of care—domains supported by established frameworks such as SPIKES (setting, perception, invitation, knowledge, empathy, and strategy) and the Calgary-Cambridge guide [9,10]. Embedding these frameworks into role‑play scenarios can promote patient-centered communication, empathy, and ethical clinical judgment before students encounter such conversations in the real world. While high‑fidelity and SP-based simulations are valuable and increasingly used, particularly for palliative and end-of-life communication, resource-sensitive peer role-play can serve as an effective stepping stone that is feasible for routine integration in busy oncology rotations.

Through this research, we aimed to illuminate the potential of role-play to strengthen the practical and interpersonal skills necessary in oncology. By assessing student outcomes in clinical skills and communication confidence, this study sought to contribute valuable insights to the development of interactive training models that better equip future oncologists to meet the complex demands of their profession.

## Methods

### Study Design and Participants

This study involved 70 graduate students majoring in oncology from Guangzhou Medical University Cancer Hospital who were in their first clinical rotation in the department of oncology from January 2022 to December 2023. The participants’ ages ranged from 22 to 24 years. Of these 70 graduate students, 36 (51%) were males and 34 (49%) were females. Using a simple randomization method, students were assigned to either a peer role-play group (n=35, 50%) or a traditional teaching group (n=35, 50%), ensuring an even distribution of gender, age, and baseline competencies.

Participants were randomized in a 1:1 ratio to the peer role‑play or traditional teaching group using a computer‑generated sequence with permuted blocks (sizes 4 and 6) stratified by gender and baseline oncology theory exam tertiles to promote balance on key variables. The randomization list was prepared by a statistician independent of the teaching and assessment teams. Allocation was concealed using sequentially numbered, opaque, sealed envelopes that were opened only after enrollment. This process yielded comparable distributions of gender, age, and baseline knowledge across groups.

Sample size was calculated based on a power analysis, which determined that a minimum of 32 students per group would be required to detect a moderate effect size (Cohen *d*=0.5) with 80% power and a 5% significance level. Both groups participated in a 3-month clinical rotation.

### Study Setting and Curricular Context

This study was conducted at Guangzhou Medical University Cancer Hospital, a tertiary cancer center in southern China. *Oncology graduate students* refer to first‑year postgraduate trainees in a 3-year Master of Medicine program who undertake supervised clinical rotations alongside coursework. Both groups participated in the standard 12‑week medical oncology rotation comprising didactic lectures, case presentations and tumor boards, ward‑based care, and outpatient clinics under faculty supervision. To accommodate the intervention while preserving clinical exposure, 1 weekly case discussion hour was replaced with a 60‑minute peer role‑play session plus structured debriefing in the role-play group; all other learning activities (lectures, case presentations, and patient care) were identical between the groups.

Before this study, structured simulation was not embedded within the oncology rotation. SPs are available at the university’s skill center primarily for preclinical communication courses; however, SP-based sessions had not been routinely implemented in the oncology rotation due to timetable and cost constraints. To introduce an accessible form of simulation, we integrated peer role‑play into the existing schedule by replacing 1 weekly case discussion hour with a structured role‑play session followed by debriefing and feedback. This approach preserved clinical exposure while creating protected time for deliberate practice of communication, clinical reasoning, and professionalism.

### Participant Characteristics and Learning Context

All participants were first‑year postgraduate students in oncology who had completed a 5-year undergraduate medical degree and were beginning their initial clinical oncology rotation; none had completed specialty residency training. Instruction and clinical care were delivered primarily in Mandarin Chinese, with Cantonese used as appropriate in-patient interactions. On the basis of institutional records, students had not received formal, oncology‑specific role‑play or simulation training before this rotation, although all had previous exposure to lecture‑based teaching and bedside observation.

Consistent with the common features of local medical education, students were accustomed to lecture‑centric learning, summative written assessments, and faculty‑led bedside teaching. Formative feedback opportunities existed but were less structured. Introducing weekly peer role‑play with facilitated debriefing was intended to provide regular, structured opportunities for practice, feedback, and reflection aligned with experiential learning principles.

### Intervention

#### Peer Role-Play Group Implementation

The peer role-play group participated in a structured role-play curriculum designed to enhance clinical skills and communication in oncology. The implementation of the role-play sessions involved several key steps.

#### Case Presentation

At the beginning of each week, the instructor conducted a case presentation session for the role-play group. During this session, the instructor introduced a real patient case, detailing the patient’s medical history, physical examination findings, diagnostic results, and the clinical reasoning behind the diagnosis. This foundation enabled students to familiarize themselves with real-life oncology cases and understand the complexities involved in patient interactions.

#### Classroom Teaching and Role-Play Instruction

Following the case presentation session, the students attended a classroom session in which the instructor introduced the principles and significance of role-play in clinical education. The instructor explained the objectives of role-play in enhancing empathy, communication skills, and diagnostic reasoning. The instructor then demonstrated how role-play scenarios would be structured, detailing each step of the role-playing process.

#### Role-Play Scenario Execution

Each week, students engaged in a 60-minute role-play session. Students were divided into small groups, with each group member assigned a specific role within the scenario: health care provider, patient, or observer. The health care provider role required students to undertake patient interactions, including history taking, explanation of diagnosis, treatment planning, and handling patient emotions. Students in the patient role were given background information to simulate the patient’s condition and emotional state, whereas observers provided feedback and noted areas for improvement.

#### Debriefing and Feedback

After each role-play session, students and instructors gathered for a structured debriefing. Instructors facilitated reflective discussions, allowing students to share their experiences and insights. Feedback was provided on communication style, empathy, clarity, and clinical decision-making. The debriefing emphasized areas in which students excelled and identified specific skills for improvement, thereby promoting a safe environment for learning and growth.

#### Traditional Teaching Group

The traditional teaching group followed the standard clinical rotation curriculum, which included lectures, case presentations, and observational learning. Students in this group participated in routine patient care activities supervised by attending physicians without structured role-play exercises.

### Assessment and Data Collection

To evaluate the effectiveness of the intervention, assessments were conducted for both groups before and after the rotation using 3 primary measures.

#### Oncology Theory Exam

A standardized written test covering key oncology knowledge areas, including diagnosis, treatment protocols, and patient management principles, was used to evaluate each student’s theoretical knowledge. The exam was administered only at the beginning of the clinical rotation (before the intervention) to assess students’ baseline knowledge in oncology. This examination was not repeated at the end of the rotation.

#### Mini-Clinical Evaluation Exercise

The Mini-Clinical Evaluation Exercise (Mini-CEX), a well-recognized tool for evaluating clinical skills [11,12], was used to assess practical competencies. The Mini-CEX was conducted only at the end of the clinical rotation, after the intervention, to evaluate the practical competencies and clinical skills of the students. Students obtained medical histories from patients and conducted physical examinations. The Mini-CEX assessed students across 7 criteria using a 9-point scale:

1. History taking: accuracy in gathering patient history, responding to nonverbal cues, and demonstrating empathy
2. Physical examination: competence in conducting examinations in an organized manner, maintaining patient privacy, and managing discomfort
3. Professionalism: respect, compassion, ethical standards, and confidentiality
4. Clinical judgment: students’ ability to select and execute appropriate diagnostic tests and weigh the risks and benefits of various treatment options
5. Physician-patient communication: clarity in explaining medical tests, obtaining consent, and educating patients
6. Organizational efficiency: skill in prioritizing patient care and effectively using resources
7. Overall competence: integration of clinical knowledge and overall patient care effectiveness

The Mini-CEX scoring encompassed “below expectations” (1-3 points), “meeting expectations” (4-6 points), and exceeding expectations (7-9 points). All assessments were conducted by a single evaluator who was blinded to the study condition of the students and was unaware of whether the assessment was before or after the intervention. This evaluator was not involved in other aspects of the study to avoid any potential bias and ensure consistency in the assessments.

#### Satisfaction Survey for the Role-Play Group

At the end of the rotation, students in the peer role-play group completed a satisfaction questionnaire. This survey measured their perceived value of the role-play activities, including aspects of engagement, realism, feedback quality, communication skill improvement, empathy development, and overall satisfaction with role-play as a learning method. The satisfaction survey comprised the following six items, each rated on a 5-point Likert scale (1=“strongly disagree”; 5=“strongly agree”): (1) “The role-play activities made me more involved in the learning process” (engagement), (2) “The scenarios in role-play felt realistic, helping me experience a clinical environment” (realism), (3) “I am satisfied with the quality of feedback received during the role-play activities” (feedback quality), (4) “I feel more confident in communicating with patients due to the role-play sessions” (communication skill improvement), (5) “The role-play exercises helped me better understand patients’ emotions and needs” (empathy development), and (6) “I am generally satisfied with the overall effectiveness of the role-play teaching method” (overall satisfaction).

To improve the validity of the satisfaction survey, it was pilot-tested with a similar cohort to refine question clarity and response consistency.

### Data Analysis

Data were analyzed to compare outcomes between the peer role-play and traditional teaching groups. Paired 2-tailed *t* tests assessed within-group improvements, and independent *t* tests compared between-group performance on the oncology theory exam and Mini-CEX. The satisfaction survey results were analyzed using descriptive statistics to summarize the feedback from the role-play group. Statistical significance was defined as *P*<.05.

### Ethical Considerations

This study was conducted in accordance with the principles of the Declaration of Helsinki. Ethics approval was obtained from the ethics committee of the Affiliated Cancer Hospital of Guangzhou Medical University (number 11/01/22). 70 first-year oncology graduate students were asked to participate in the study. All of them had signed informed consent prior to participation. All data were pseudonymized before being subjected to statistical analysis. Participants did not receive any financial compensation. As an incentive, the 5 best-performing students in each group were awarded a book prize.

## Results

### Baseline Theory Exam Scores

At baseline, the average theory exam scores were comparable between the 2 groups. The peer role-play group achieved a mean score of 91.12 (SD 2.15), whereas the traditional teaching group scored an average of 91.33 (SD 2.16). Statistical analysis indicated no statistically significant difference between the groups’ baseline theoretical knowledge (*P*>.05), confirming a similar level of theoretical understanding at the start of the rotation.

### Mini-CEX Assessment

All trainees completed the Mini-CEX evaluation within an average of 36 (SD 0.5) minutes, and post-evaluation feedback required approximately 6.6 (SD 0.4) minutes per student. After the rotation, the peer role‑play group performed significantly better than the traditional teaching group on the Mini‑CEX domains of physician-patient communication, history taking, clinical judgment, and overall clinical competence (*P*<.05 in all cases). Detailed Mini-CEX scores for both groups are provided in Table 1.

**Table 1:** The distribution of the scale scores on the Mini-Clinical Evaluation Exercise assessment in the 2 groups.

Item		Peer role-play group (n=35), n (%)	Traditional teaching group (n=35), n (%)		*P* value	
**Medical history taking**						.03
	Meets expectations	13 (37)	23 (66)			
	Exceeds expectations	22 (63)	12 (34)			
**Clinical judgment**						.02
	Below expectations	0 (0)	2 (6)			
	Meets expectations	15 (43)	24 (69)			
	Exceeds expectations	20 (57)	9 (26)			
**Physician-patient communication**						.004
	Meets expectations	12 (34)	25 (71)			
	Exceeds expectations	23 (66)	10 (29)			
**Professionalism**						.03
	Meets expectations	14 (40)	24 (69)			
	Exceeds expectations	21 (60)	11 (31)			
**Physical examination**						.81
	Meets expectations	18 (51)	16 (46)			
	Exceeds expectations	17 (49)	19 (54)			
**Organizational effectiveness**						.34
	Meets expectations	16 (46)	21 (60)			
	Exceeds expectations	19 (54)	14 (40)			
**Overall capabilities**						.03
	Meets expectations	12 (34)	22 (63)			
	Exceeds expectations	23 (66)	13 (37)			

### Satisfaction Survey Results for the Role-Play Group

At the end of the study, students in the peer role-play group completed a satisfaction survey to assess their perceptions of the role-play experience (Table 2). Results indicated high satisfaction levels, with most students agreeing that role-play was a valuable and engaging learning tool. Key areas of positive feedback included engagement (32/35, 91% agreement and “strongly agree”), the realism of the scenarios (29/35, 83% agreement and “strongly agree”), the quality of the feedback received (32/35, 91% agreement and “strongly agree”), and overall satisfaction (33/35, 94% agreement and “strongly agree”).

**Table 2:** Students’ satisfaction evaluation in the role-play group (n=35)^a^.

	Strongly disagree, n (%)	Disagree, n (%)	Neutral, n (%)	Agree, n (%)	Strongly agree, n (%)
Engagement	1 (3)	0 (0)	2 (6)	25 (71)	7 (20)
Realism	2 (6)	2 (6)	2 (6)	24 (69)	5 (14)
Communication skill improvement	1 (3)	0 (0)	1 (3)	24 (69)	8 (23)
Feedback quality	0 (0)	1 (3)	1 (3)	28 (80)	4 (11)
Empathy development	0(0)	1(3)	2(6)	29(83)	3(8)
Overall satisfaction	1 (3)	1 (3)	0 (0)	26 (74)	7 (20)

^a^1=strongly disagree, 2=disagree, 3=neutral, 4=agree, and 5=strongly agree.

## Discussion

This study examined the effectiveness of peer role-play versus traditional teaching methods in enhancing the clinical competencies among oncology graduate students. The findings offer valuable insights into the role of interactive learning methods, particularly in fostering communication skills and clinical decision-making.

The results indicate that, while both groups had similar theoretical knowledge at baseline, the peer role-play group exhibited significantly higher scores in practical skills, including physician-patient communication, medical history taking, and overall clinical competence as assessed using the Mini-CEX (*P*<.05). This aligns with previous studies suggesting that role-play is an effective tool for enhancing soft skills in medical education, especially communication and empathy [13,14]. These improvements are crucial for oncology care, where patient interactions often involve delivering complex news and managing emotional responses.

Moreover, the peer role-play group demonstrated superior clinical decision-making skills, suggesting that role-play scenarios may foster critical thinking and integrative skills by actively engaging students in problem-solving rather than passive observation [15]. This active learning approach likely provides students with a deeper understanding of clinical workflows, which is essential for patient-centered care in high-stakes oncology settings.

The high levels of satisfaction reported by students in the role-play group underscore its acceptability and perceived value in clinical education. Students valued the realism of the scenarios and the opportunity for constructive feedback, contributing to the observed improvements in communication and clinical skills. These positive feedback results suggest that role-play was well received by students, particularly in terms of engagement and the quality of feedback. However, the results do not directly suggest an enhancement of learning outcomes or motivation, which would require further investigation.

Our scenarios deliberately targeted competencies central to oncology and palliative care communication—eliciting patient values and concerns, conveying difficult information, and negotiating shared decision‑making—guided by the SPIKES framework and principles from the Calgary-Cambridge guide [9,10]. Prior work has shown that structured role‑play and simulation can improve learners’ confidence and observable communication behaviors in serious illness conversations, whereas ongoing feedback and deliberate practice are critical to maintaining gains. In settings in which SP programs and high‑fidelity simulations are not yet routinely available in oncology rotations, peer role‑play provides a pragmatic, low-cost modality that can be delivered regularly, reinforced by structured debriefing. Future work at our institution will extend scenario content to explicitly include dialogues on end of life and goals of care and compare peer role‑play with SP-based approaches on performance and transfer to clinical practice.

Despite promising findings, several limitations should be acknowledged. First, this study was conducted at a single institution and focused solely on oncology graduate students, which may limit its generalizability to other medical fields. Additionally, the short rotation duration raises questions about the long-term retention of skills acquired through role-play. Another limitation is the potential for evaluator bias in Mini-CEX assessments, although the evaluator was blinded to group assignments.

This single‑center study used a posttest-only design for the Mini‑CEX and administered the theory exam only at baseline. Consequently, we cannot estimate within‑group change or link knowledge gains to observed performance. We also relied on a single blinded assessor for Mini‑CEX ratings, which may limit the generalizability of judgments. Satisfaction data were self‑reported and limited to the role‑play group. Finally, we did not include a dedicated empathy instrument or teamwork measure.

Future research could address these limitations by expanding sample sizes, including multiple institutions, and assessing long-term skill retention. Incorporating objective measures of patient outcomes could help assess the real-world impact of role-play on patient care quality. Additionally, comparing role-play with other interactive learning methods such as simulation-based training could deepen understanding of the most effective approaches for teaching clinical skills.

In conclusion, this study demonstrates that peer role-play is an effective and well-received method for enhancing clinical competencies in oncology education. By engaging students in realistic scenarios, role-play fosters key skills in communication, decision-making, and teamwork, which are all crucial for patient-centered care. These findings support the integration of role-play into clinical curricula as a valuable complement to traditional teaching methods, especially in fields that demand strong interpersonal and decision-making skills.

## Data Availability

All datasets generated for this study were included in the manuscript.

## Abbreviations

Mini-CEX: Mini-Clinical Evaluation Exercise
SP: standardized patient
SPIKES: setting, perception, invitation, knowledge, empathy, and strategy
